# Pilot-Scale Production of Chito-Oligosaccharides Using an Innovative Recombinant Chitosanase Preparation Approach

**DOI:** 10.3390/polym13020290

**Published:** 2021-01-18

**Authors:** Chih-Yu Cheng, Chia-Huang Tsai, Pei-Jyun Liou, Chi-Hang Wang

**Affiliations:** Department of Marine Biotechnology, National Kaohsiung University of Science and Technology, Kaohsiung 81157, Taiwan; Chronotsai@genovior.com.tw (C.-H.T.); Pei-JyunLiou@eurofins.com (P.-J.L.); f16plant@gmail.com (C.-H.W.)

**Keywords:** chitosanase, selective precipitation, chito-oligosaccharides, recombinant protein

## Abstract

For pilot-scale production of chito-oligosaccharides, it must be cost-effective to prepare designable recombinant chitosanase. Herein, an efficient method for preparing recombinant *Bacillus* chitosanase from *Escherichia coli* by elimination of undesirable substances as a precipitate is proposed. After an optimized culture with IPTG (Isopropyl β-d-1-thiogalactopyranoside) induction, the harvested cells were resuspended, disrupted by sonication, divided by selective precipitation, and stored using the same solution conditions. Several factors involved in these procedures, including ion types, ionic concentration, pH, and bacterial cell density, were examined. The optimal conditions were inferred to be pH = 4.5, 300 mM sodium dihydrogen phosphate, and cell density below 10^11^ cells/mL. Finally, recombinant chitosanase was purified to >70% homogeneity with an activity recovery and enzyme yield of 90% and 106 mg/L, respectively. When 10 L of 5% chitosan was hydrolyzed with 2500 units of chitosanase at ambient temperature for 72 h, hydrolyzed products having molar masses of 833 ± 222 g/mol with multiple degrees of polymerization (chito-dimer to tetramer) were obtained. This work provided an economical and eco-friendly preparation of recombinant chitosanase to scale up the hydrolysis of chitosan towards tailored oligosaccharides in the near future.

## 1. Introduction

Chitosanases (EC 3.2.1.132) catalyze the hydrolysis of β-1,4 glycosidic linkages in chitosan, a deacetylated derivative of chitin extracted from an abundant source of shellfish exoskeletons. This polysaccharide and its derivatives have diverse applications in science, medicine, and the cosmetic industry [1]. Chito-oligosaccharides have received a great deal of attention owing to their interesting biological properties such as their inhibitory effects on the growth of microorganisms and tumor cells [2,3]. Furthermore, they are able to induce the expression of disease-resistance-response genes in higher plants [4,5]. These properties suggest the potential for chito-oligosaccharides and their degrading enzyme, chitosanase. The production of chito-oligosaccharides by enzymatic hydrolysis has been reported previously [6]. Moreover, because of the high cost of commercial chitosanase, studies have been focused on several enzyme immobilization recycling techniques [7,8], increasing the enzymatic concentration through stepwise substrate addition [7], continual production in a dual reactor system [9,10], and the use of cheap, crude, non-specific enzymes [11,12].

Chitosanases have been purified from several organisms such as viruses [13], bacteria [14], fungi [15], and plants [16] and classified into glycoside hydrolase families 3, 5, 7, 8, 46, 75, and 80 [17]. Among these, family 46 has been extensively characterized [18], particularly *Bacillus* chitosanases which have been investigated by several aspects [10,19,20,21,22,23,24,25]. To obtain these purified enzymes, multiple purification steps often are necessary and the process routinely entails a combination of ammonium sulfate precipitation and chromatographic techniques such as anion exchange and gel filtration [14,26]. The gene encoding the enzyme has been cloned, and under certain circumstances, recombinant chitosanase has been obtained through one-step purification. For instance, an affinity-based method using a fusion His-tagged protein, which exhibits a high affinity towards immobilized metal affinity chromatography, is quite useful [25]. Only few studies on chitosanase produced from *Bacillus* through one-step purification have been conducted [20]. Nevertheless, the possibility of heavy metal leaching, removal of the His-tag, and chromatography resin savings tend to be the major concerns limiting the application of this process. 

Among the routine purification methods including extraction, precipitation, ultracentrifugation, and chromatography, precipitations are extensively used as a bulk purification method in industry. Proteins are fractionated through the addition of salts or organic solvents, and their solubility is modulated based on changes in pH and temperature [24]. Ammonium sulfate is the most common precipitant for salting out of proteins. Unfortunately, along with the target protein, several other undesired proteins are also precipitated through this technique. Hence, methods that apply a combination of precipitants have been developed to enhance their specificity. The use of trichloroacetic acid and acetone [27], affinity precipitation using a macroaffinity ligand [28], selective precipitation using oligovalent ligands and ammonium sulfate [29], and the combination of precipitation with a two-phase extraction system [22,28] are some of the combination methods that have been explored. However, the scope and specificity of these methods are influenced by the properties of the target proteins. 

A chitosanase from *Bacillus circulans* MH-K1 (Csn) (GH-46) hydrolyzed chitosan in an endo-splitting manner and released (GlcN)_2_, (GlcN)_3_, and (GlcN)_4_ in almost equivalent proportions. The enzyme composed of 259 amino acids [19] has been expressed in *E. coli* [30] to facilitate the investigation of molecular engineering for their hydrolysis products. Herein, an efficient purification process for this recombinant chitosanase by removal of undesirable substances as a precipitate is proposed. The effects of several factors on the selective precipitation including pH, chemical species, buffer concentration, and bacterial suspension concentration were investigated. Moreover, the purified chitosanase was applied for the preparation of 500 g of chito-oligosaccharide.

## 2. Materials and Methods 

### 2.1. Bacterial Strains and Plasmids

Vector pET22b containing the *Bacillus circulans* MH-K1 chitosanase gene (*csn*, complete coding sequence NCBI accession number D10624.2) [30] was supplied by Professor Li (Department of Applied Chemistry, National Chiao Tung University, Hsinchu, Taiwan). The vector was double-digested with *Nde*I and *Xho*I restriction enzymes, and the insert DNA fragment (*csn*; approximately 1.0 kb) was ligated into pET20b. The resultant clone, pET20b-*csn*, was transformed into *Escherichia coli* strain BL21 (DE3) for recombinant protein expression.

### 2.2. Culture Conditions

A single colony of *E. coli* strain BL21 (DE3) harboring the plasmid pET20b-*csn* was inoculated in 5 mL of Luria–Bertani (LB) medium containing 0.1 mg/mL ampicillin (LB/Amp) and was cultivated at 37 °C. The overnight-grown bacterial cells (5 mL) were then transferred into a 500-mL baffled flask containing 50 mL of LB medium supplemented with ampicillin (0.1 mg/mL) and IPTG (Isopropyl β-d-1-thiogalactopyranoside) (1 mM), followed by incubation at 37 °C for 2.5–5 h until an OD_600nm_ of 2.0 was attained.

### 2.3. Enzyme Purifications

All purification steps were performed at ambient temperature (25 °C) unless otherwise specified. The cell pellet was obtained by centrifugation (8400× *g*, 10 min, 4 °C) and resuspended in different buffers (phosphate, acetate, and citrate) of various pH values (3.0–8.0) and molarities (20–800 mM). By controlling the added volumes of suspension buffer, the resulting cell density by turbidity measurement of the bacterial suspension was adjusted to equivalents of OD_600nm_ from 20 to 160. A typical suspension buffer used for the control sample was 20 mM phosphate buffer with a pH of 7.0, for a resulting concentration of OD_600nm_ 2.0. The cells were then disrupted by sonicating for 10 min (on: 0.5 s; off: 1.0 s; output power: 100 W) (BRANSON, Digital Sonfier^®^, Missouri, TX, USA) in an ice bath. After being allowed to stand for 4 h at 4 °C, the supernatant (S; the soluble protein fraction) was obtained through centrifugation (8400× *g*, 20 min, 4 °C) and analyzed directly. The precipitate was washed twice with a similar buffer and resuspended in phosphate buffer (pH = 7.0, 20 mM) for performing the assay. At least 3 replicates for each procedure were performed.

In an alternative approach, the soluble protein fraction of the aforementioned control sample was applied onto an anion-exchanged chromatographic column (HiTrapTM in 5 mL of Q-Sepharose; Amersham Biosciences, Buckinghamshire, England, UK). The column was pre-equilibrated with phosphate buffer (pH 7.0, 20 mM) and eluted with a linear gradient of NaCl (10–100 mM) using a similar buffer at a flow rate of 2 mL/min. The fractions exhibiting chitosanase activity were pooled, concentrated (GE Healthcare, Vivaspin 20; 5 kDa molecular weight cut-off, Chicago, IL, USA), and stored at 4 °C for further analyses. 

### 2.4. Chitosanase Activity Assay

Chitosan powder used in this study was 90% deacetylated, 200–500 kD, over 120 mesh and obtained from a local supplier in Taiwan (Charming & Beauty Co., Taipei, Taiwan). Chitosanase activity was analyzed by estimating the amount of reducing sugars according to the dinitrosalicylic acid (DNS) method [31]. In this assay, chitosan (0.1 mL, 1%, pH = 6.0) was mixed with the enzyme (0.1 mL), suitably diluted in phosphate buffer (20 mM, pH = 7.0), and incubated for 30 min at 37 °C to allow enzymatic hydrolysis. The DNS reagent (0.2 mL) was then added, and the resulting mixture was boiled for 15 min, chilled, and centrifuged to isolate the insoluble chitosan. The resulting adducts (0.1 mL) of reducing sugars were measured spectrophotometrically at 543 nm (CHAMELEON microplate reader; HIDEX, Turku, Finland). Their absorption coefficient was determined to be 141 M-1 when d-glucosamine served as the control sample. One unit of chitosanase activity (U) is defined as the amount of enzyme required to release 1 μmol of detectable reducing sugars in 1 min at 37 °C [15]. 

### 2.5. Determination of Protein Concentration

The protein concentration was determined using a Bradford assay (Bio-Rad Laboratories, Inc., Hercules, CA, USA) with bovine serum albumin as the standard. Furthermore, the protein was subjected to sodium dodecyl sulfate polyacrylamide gel electrophoresis (SDS-PAGE) and visualized by staining with Coomassie Brilliant Blue G-250. The quantitative image analysis was performed using BioRad Image Lab Version 4.1 (Bio-Rad Laboratories, Inc., Hercules, CA, USA).

### 2.6. Bulk Preparation of Chitosanase 

A single colony of *E. coli* strain BL21 (DE3) harboring the plasmid pET20b-*csn* was inoculated into LB/Amp medium (5 mL) and incubated at 37 °C. After culturing overnight, it was transferred into 50 mL of LB/Amp medium and incubated at 37 °C. Once the cell density reached 2.5 at 600 nm, the culture was transferred into 500 mL of LB/Amp/IPTG and incubated at 37 °C to reach an OD of 2.0 at 600 nm. Later, the cell pellet was obtained by centrifugation (8400× *g*, 10 min, 4 °C), resuspended in 25 mL of 300 mM sodium dihydrogen phosphate, and disrupted through sonication for 25 min in an ice bath. After allowing it to stand at 4 °C for longer than 4 h, the lysate was centrifuged at 8400× *g* for 20 min at 4 °C, and the supernatant consisting of soluble protein fractions was obtained. The purified enzyme was stored at 4 °C until its use in the pilot-scale production of chito-oligosaccharides. 

### 2.7. Thin-Layer Chromatographic Analysis of Enzymatic Hydrolysate

Chito-oligosaccharides were analyzed qualitatively through thin-layer chromatography (TLC). The oligosaccharides were developed using a solvent system of n-propyl alcohol and 28% ammonium water (2:1, *v*/*v*) at 55 °C on silica gel 60 F254 25 aluminum sheets (Merck, Darmstadt, Germany). Sugar spots on the sheet were visualized by spraying 0.1% ninhydrin and baking at 100 °C for 10–15 min.

### 2.8. Pilot-Scale Production of Chito-Oligosaccharides 

A 500 g batch reaction was performed by adding approximately 2500 units of semi-purified chitosanase to a 10 L chitosan solution (5%) at ambient temperature for 72 h. The reaction mixture was prepared by suspending 500 g of chitosan and an appropriate amount of chitosanase in 9.65 L of water. Later, 350 mL of acetic acid was added with vigorous agitation. A nearly clear and viscous solution with a pH of 5.5–6.0 was obtained. Due to the high viscosity of 5% chitosan, the reaction solution was proposed to stand without agitation at ambient temperature and then stirred thoroughly before sampling. The efficiency and products of enzymatic hydrolysis were then analyzed by DNS assay and TLC, respectively.

## 3. Results and Discussions

### 3.1. Enzyme Expression and Induction 

In order to stabilize the plasmid, increase the protein expression, and facilitate subsequent gene mutations, the chitosanase gene of pET22b-*csn* [30] was transposed to a smaller plasmid, pET20b. As a result, the recombinant chitosanase was composed of 260 amino acids, including one from the start codon of pET20b and 259 from the mature *Bacillus* MH-K1 chitosanase [19]. For the enzyme production, the recombinant *E. coli* cells were cultivated in LB/Amp medium and then transferred to LB/Amp/IPTG medium to induce gene expression. IPTG induction was performed in larger-volume flasks with a high surface-area-to-volume ratio to increase the surface aeration rate for at least 2 h. According to our 2008 study [30], recombinant chitosanase is expressed intracellularly and is hence found in the soluble fraction of the cell lysate when harvested recombinant *E. coli* cells are suspended in 20 mM phosphate buffer with a pH of 7.0 and disrupted by sonication. To reduce the steps required for protein purification, we examined the use of a multifunctional solution (MF solution) as a suspension buffer, precipitating agent, and storage buffer. In this study, for example, 20 mM phosphate buffer with a pH of 7.0 was used as the MF solution for the control sample to resuspend the cell pellet, assist cell lysis, precipitate the undesired proteins, and provide suitable storage conditions. 

### 3.2. Effect of Chemical Species on Protein Precipitation

**Figure 1:**
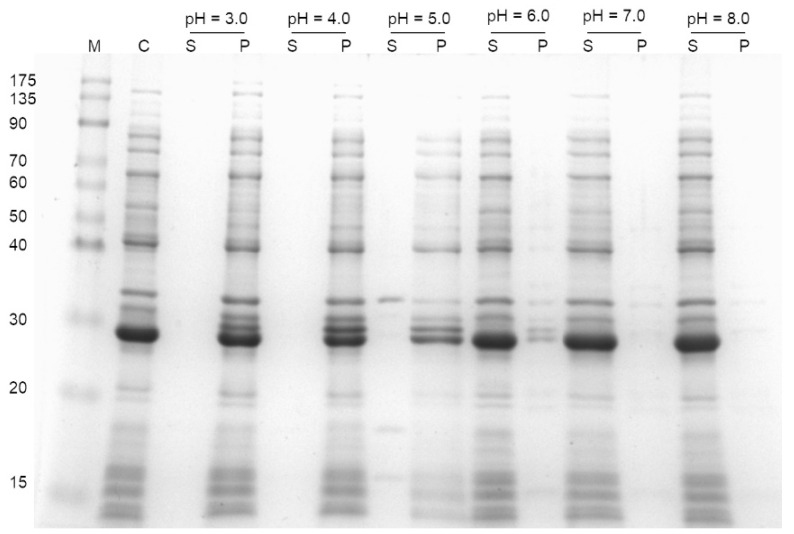
SDS-PAGE analysis displaying the pH effects of universal buffer (citrate-phosphate buffer) on protein precipitation. After suspension in universal buffer with varying pH, sonication, and centrifugation, the resulting supernatant (S) and pellet (P) were assayed. The control sample was treated using 20 mM phosphate buffer with a pH of 7.0. M: protein marker; C: control.

The universal buffer (McIlvaine buffer, also known as citrate-phosphate buffer), a two-component mixture comprising 0.3 M Na_2_HPO_4_ and 0.01 M citric acid, with a pH in the range of 3–8, was initially used as the MF solution. After the cell pellet was resuspended in these universal buffers, sonicated, and centrifuged, the resulting supernatant and pellet were assayed (Figure 1). A major band around 30 kDa corresponding to the *Bacillus* MH-K1 chitosanase was discernable in the pellet fractions (P) at low pH (3–5) and in the supernatant fractions (S) at high pH (6–8). A minor selective precipitation, which fractionalized several undesired proteins into opposite fractions, occurred when the pH was 5 (Figure 1, lane 3S). However, one or two additional bands appeared at approximately 30 kDa above the chitosanase band, at low pH (lanes 1P, 2P, and 3P). This could be attributed to citric acid causing protein modification [32,33]. Nevertheless, additional experiments are needed to clarify this observation. 

Acetate buffer was also used as the MF solution to assess the precipitation performance. However, the crude extract proteins were all precipitated when acetate buffer with a pH of 3 or 4 was used. Conversely, when pH = 5 buffer was used, the whole proteins presented in the supernatant fraction, i.e., no precipitation occurred (data not shown). Explicitly, no selective precipitation was observed when using the acetate buffer system.

### 3.3. Effect of pH on Protein Precipitation

**Figure 2:**
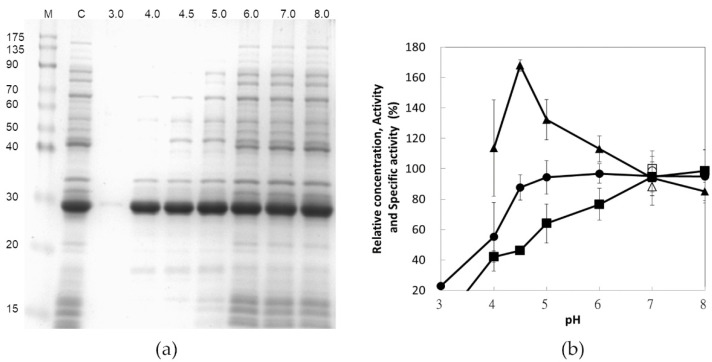
Effects of phosphate buffer pH on selective precipitation, represented using SDS-PAGE (**a**) and a run chart (**b**). The resulting supernatants were assayed for their SDS-PAGE purity (**a**), protein concentration (■), enzyme activity (●), and specific activity (▲) relative to the control sample (□, ○, and △). M: protein marker; C: control; 3.0–8.0 represents the pH value of the phosphate buffer employed.

For eliminating the reaction of citric acid, 300 mM phosphate buffer alone was tested for its selective precipitation at various pH values and the resulting supernatant fraction is depicted in Figure 2a. Notably, the lower the pH value, the lower the amount of protein presented in SDS-PAGE. However, the strength of the major band representing chitosanase was consistent until the pH reached 3.0 (Figure 2a, lane 1). The quantitative analysis of chitosanase activity and total protein concentration shown in Figure 2b were consistent with the SDS-PAGE analysis, except at pH = 4. At this pH value, there was no decrease in the amount of chitosanase (Figure 2a, lane 2), but 40% of the enzymatic activity was lost (Figure 2b). This suggests an irreversible denaturation of chitosanase. This undefined situation combined with the low capacity of phosphate buffer around the pH of 4 might have contributed to a higher experimental deviation. For pH higher than 8, chitosanase would become precipitated (data not shown). Hence, the optimum specific activity (about 40 U/mg) of chitosanase was obtained at the pH of 4.5. In general, selective precipitation recovers the majority of target proteins as precipitate. On the contrary, this procedure precipitates undesirable substances and the recombinant chitosanase remains as a soluble state in the supernatant, which prevents the risks of target enzyme denaturing due to precipitation. 

### 3.4. Effect of Buffer Concentration on Protein Precipitation

**Figure 3:**
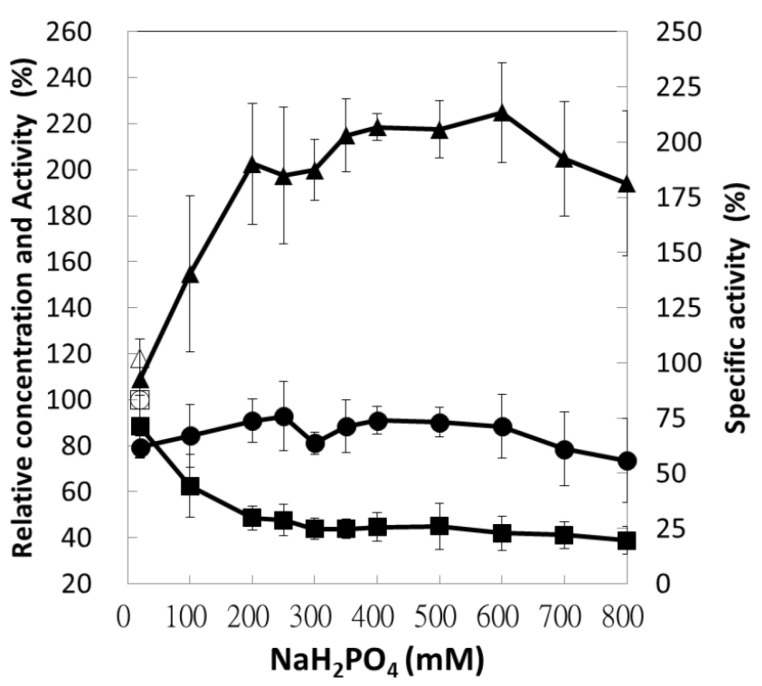
Concentration effects of sodium dihydrogen phosphate on selective precipitation. The supernatants were assayed for their protein concentration (■), enzyme activity (●), and specific activity (▲) relative to the control sample (□, ○, and △). In this experiment, the OD_600nm_ value of bacterial suspension was 2.0.

Because the phosphate buffer yielded optimal precipitation at the pH of 4.5, which is similar to the pH of NaH_2_PO_4_ solution, NaH_2_PO_4_ was used directly as the MF solution. The concentration effect of NaH_2_PO_4_ on selective precipitation is represented in Figure 3. As the concentration was increased from 0 to 200 mM, the protein amount was reduced through precipitation, and an equilibrium was achieved at a concentration higher than 200 mM; however, a better stabilization was achieved at above 300 mM, which was taken to be the optimal concentration of the MF solution. Enzyme activity, protein concentration, and specific activity tended to slightly decline when the concentration exceeded 600 mM.

### 3.5. Effect of Bacterial Suspension Concentration

**Figure 4:**
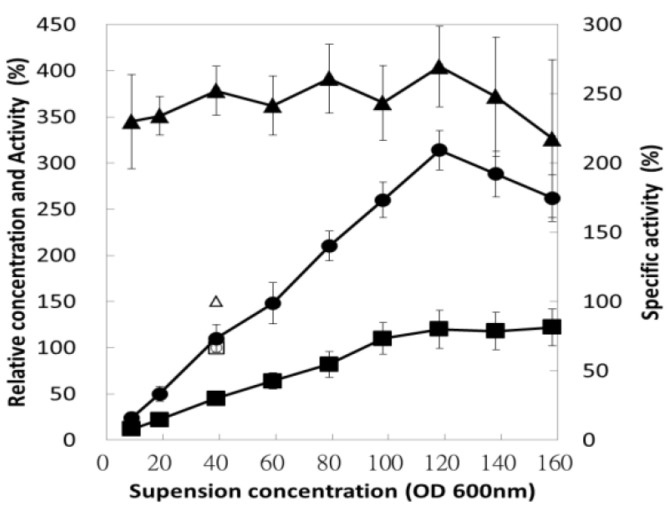
Effects of bacterial suspension concentration on selective precipitation. The bacterial suspension was diluted to an appropriate concentration with phosphate buffer (300 mM, pH = 4.5). After selective precipitation, the resulting supernatants were assayed for their protein concentration (■), enzyme activity (●), and specific activity (▲) relative to the control sample (□, ○, and △). The control sample was suspended in 20 mM phosphate buffer with a pH of 7.0 and the OD_600nm_ was 40.

The cell density used during purification has a direct influence on the industrial process because it determines the working volume and the operating unit in a downstream process. Operating at a higher density saves space, time, and expenses. However, a high ratio of biomass to suspension buffer always results in poor lysis and poses operational difficulties. Therefore, after the harvested cell was resuspended, the bacterial suspension was diluted with a suitable amount of MF solution (300 mM NaH_2_PO_4_) to an appropriate concentration. As inferred from Figure 4, for a cell density equivalent to OD_600nm_ < 100, enzyme activity and protein concentration were strongly related to the cell density; these resulted in a negligible influence on the specific activity of the enzyme. Moreover, the majority of the *E. coli* proteins were removed through precipitation, but chitosanase remained. Consequently, once the equivalent OD_600nm_ value of the bacterial suspension exceeded 100, operation became difficult due to its high viscosity, which affects the efficiency of sonication and enzyme recovery, even under carefully controlled conditions. 

### 3.6. Bulk Preparation of Chitosanase 

**Figure 5:**
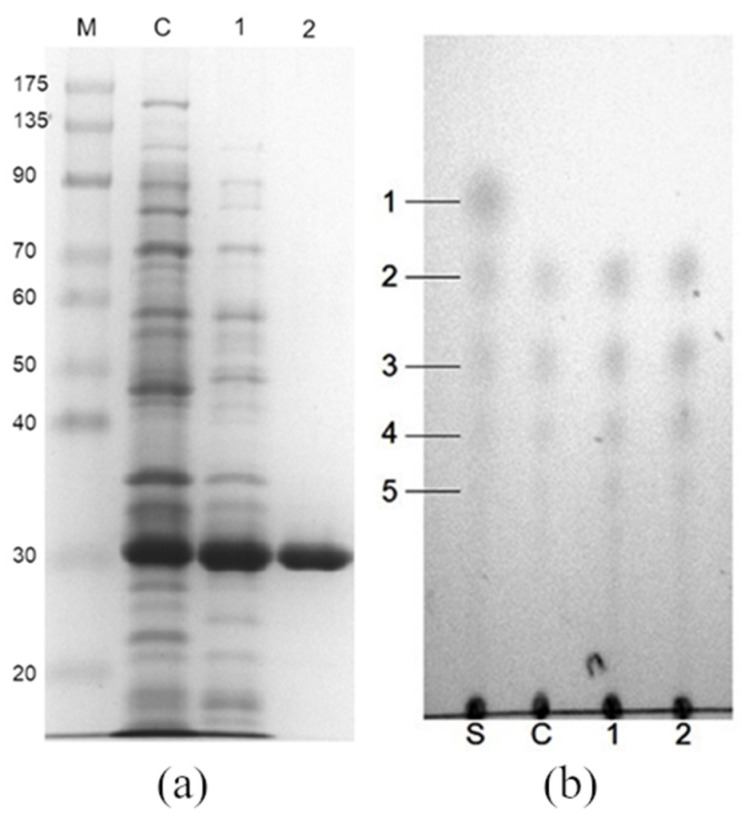
SDS-PAGE (**a**) and thin-layer chromatography (TLC) (**b**) analysis of purified chitosanase and hydrolysates. C: Crude extract; 1: chitosanase purified through selective precipitation; 2: chitosanase purified through fast protein liquid chromatography (FPLC); M: protein marker; S: chito-oligosaccharide standards with number of monomers ranging from 1 to 5.

The optimal precipitation condition involved the addition of 300 mM NaH_2_PO_4_ to suspend the bacterial cells, yielding a cell density equivalent to OD_600nm_ lower than 120. Chitosanase isolated through the selective precipitation of 500 mL of the bacterial culture was compared with the crude extract and the purified chitosanase; the results are presented in Figure 5a and Table 1. The crude extract and the suspension of the control sample were processed in 20 mM phosphate buffer with a pH of 7.0. High-homogeneity chitosanase (>95%) was obtained through fast protein liquid chromatography (FPLC) purification using an anion-exchange chromatographic column with 40 mM NaCl as the eluent. The production and purification efficiencies are compared in Table 1, which indicates that the chitosanase was purified by 1.8-fold, with 89% activity recovery only through selective precipitation. The homogeneity of the bulk preparation of chitosanase (Figure 5a) is lower than that of little preparation (Figure 2a) as a result of the reducing efficiency of centrifugation owing to the large bulk. However, the homogeneity is above 70%, as revealed by the data in Table 1 (42/57 = 74%) and SDS-PAGE (Figure 5a) (70–80%) calculated through quantitative image analysis (BioRad, Image Lab Version 4.1). 

The chitosan hydrolysates obtained using the crude enzyme and purified chitosanase (selective precipitation and FPLC) were similar, with multiple degrees of polymerization from chito-dimer to pentamer, as shown in Figure 5b. For industrial applications, it is beneficial to obtain a catalyst without any purification steps required to remove contaminants. However, the risks posed by the presence of contaminants in long-term enzymatic reactions, such as destroying the enzyme itself or consuming the products, should also be admitted. Fortunately, throughout this precipitation process (by 300 mM NaH_2_PO_4_, pH = 4.5), several undesirable substances including proteins and their enzyme activities were removed and abandoned, respectively. For example, inappropriate digestion is prevented because of the inhibition of potassium ions (>1 mM) on the extra protease of *E. coli* [34]. Besides, this buffer also provided an extreme storage condition (pH = 4.5) which minimizes contamination risk. When chitosanase was stocked within this buffer at 4 °C, 80% of its activity was retained for a minimum of 4 weeks. For its use in industry preparations, these properties should also be considered.

### 3.7. Pilot-Scale Production of Chito-Oligosaccharides

**Figure 6:**
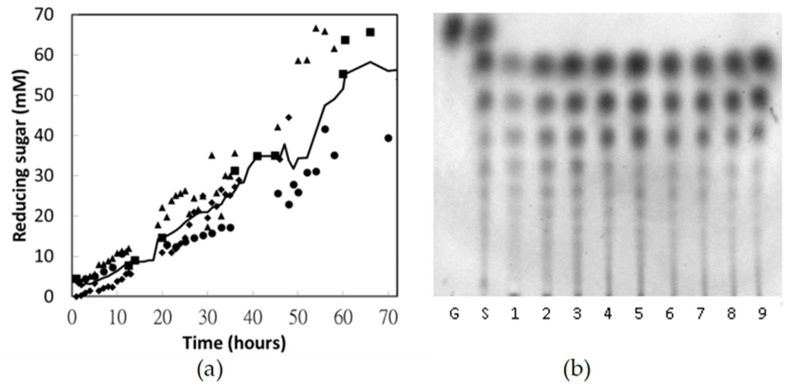
Batch reaction of chitosan hydrolysis by chitosanase purified through selective precipitation. The two methods for tracing the enzymatic reaction were dinitrosalicylic acid (DNS) assay (**a**) and TLC (**b**). (**a**) Solid line: moving average of four batch reactions, which are indicated by filled squares, diamonds, triangles, and circles. (**b**) Lane G: glucosamine; lane S: chito-oligomer standard; lanes 1–9: hydrolysates after 8-, 16-, 24-, 32-, 40-, 48-, 56-, 64-, and 72-h reaction, respectively.

A 10 L batch reactor with 5% chitosan was hydrolyzed for 72 h using the chitosanase purified through selective precipitation. The hydrolysate and time-course of the hydrolytic reaction are analyzed in Figure 6. As typical endo-splitting glycoside hydrolases, several oligosaccharides larger than (GlcN)_2_ were produced simultaneously at the primary stage (Figure 6b, lanes 1–3). After 32 h, longer oligosaccharides were hydrolyzed and (GlcN)_2_, (GlcN)_3_, and (GlcN)_4_ became the major products (Figure 6b, lanes 6–9). After 72 h, hydrolysate equivalent to 60 ± 16 mM reducing sugar was obtained. In other words, these chito-oligosaccharides have molar masses of 833 ± 222 g/mol and an average degree of polymerization of 4.7 ± 1.2 mers. A mixed product consisting of (GlcN)_2_ and (GlcN)_3_ was not obtained until several days of reaction due to the limited hydrolytic ability of *Bacillus* chitosanase against (GlcN)_4_. The high experimental deviations among the four batch reactions could be attributed to various ambient temperatures and pH values, which were influenced by the polymerization of the commercial chitosan. Hence, to obtain a certain distribution of the hydrolysate, timely detection of the enzymatic hydrolysis—for example, by TLC analysis—is recommended. 

## 4. Conclusions

An approach involving selective precipitation is established for chitosanase preparation in a cost-efficient way. An acidic condition, provided by 0.3 M NaH_2_PO_4_, was verified to precipitate most intracellular proteins within *E. coli* except recombinant chitosanase. It also provides a protective shield to avoid contamination during storage. This procedure is responsible for a green and sustainable design without hazardous substances, decreasing waste formation and reducing water consumption. It also minimizes the overall cost by providing a shorter operation time, cheaper ingredients, and higher recovery of enzyme activity than most programs targeted on recombinant protein. However, we recommend that this procedure should be conducted only for acid-proof enzymes. This approach not only improves the applicability of chitosanase for industrial chitosan hydrolysis but also allows earlier screening of designed chitosanase expressed in *E. coli* for industrial purposes or tailored polysaccharide production, which will expand the development of chitosan dramatically.

## Figures and Tables

**Table 1:** Production and purification efficiency in 0.5 L culture medium.

Step	Total Protein (mg)	Total Activity (U)	Specific Activity (U/mg)	Yield (%)	Purification Fold
Crude extract ^a^	109	2507	23	100	1.0
Selective precipitation	53	2226	42	89	1.8
FPLC	37	2109	57	84	2.5

a: Crude extract was derived from the control sample.

## Data Availability

The data that support the findings of this study are available from the corresponding author upon reasonable request.
